# Serum NY-ESO-1 antibody as a predictive biomarker for postoperative recurrence of gastric cancer: a multicenter prospective observational study

**DOI:** 10.1038/s41416-023-02540-3

**Published:** 2024-02-07

**Authors:** Takuro Saito, Yukinori Kurokawa, Kazumasa Fujitani, Ryohei Kawabata, Atsushi Takeno, Jota Mikami, Shunji Endo, Jin Matsuyama, Yusuke Akamaru, Masashi Hirota, Kentaro Kishi, Shinya Urakawa, Kei Yamamoto, Koji Tanaka, Tsuyoshi Takahashi, Mikio Oka, Hisashi Wada, Hidetoshi Eguchi, Yuichiro Doki

**Affiliations:** 1 Department of Gastroenterological Surgery, Osaka University Graduate School of Medicine, Suita, Japan; 2 Department of Gastroenterological Surgery, Osaka General Medical Center, Osaka, Japan; 3 Department of Surgery, Osaka Rosai Hospital, Sakai, Japan; 4 Department of Surgery, Kansai Rosai Hospital, Amagasaki, Japan; 5 Department of Surgery, Sakai City Medical Center, Sakai, Japan; 6 Department of Surgery, Higashi-Osaka Medical Center, Higashi-Osaka, Japan; 7 Department of Surgery, Yao Municipal Hospital, Yao, Japan; 8 Department of Surgery, Ikeda City Hospital, Osaka, Japan; 9 Department of Surgery, Toyonaka Municipal Hospital, Toyonaka, Japan; 10 Department of Surgery, Osaka Police Hospital, Osaka, Japan; 11 Department of Immuno-Oncology, Kawasaki Medical School, Okayama, Japan; 12 Department of Clinical Research in Tumor Immunology, Osaka University Graduate School of Medicine, Osaka, Japan

**Keywords:** Tumour biomarkers, Gastric cancer, Humoral immunity, Translational immunology

## Abstract

**Background:**

No reliable marker has been identified to predict postoperative recurrence of gastric cancer. We designed a clinical trial to investigate the utility of serum NY-ESO-1 antibody responses as a predictive marker for postoperative recurrence in gastric cancer.

**Methods:**

A multicenter prospective study was conducted between 2012 and 2021. Patients with resectable cT3-4 gastric cancer were included. Postoperative NY-ESO-1 and p53 antibody responses were serially evaluated every 3 months for 1 year in patients with positive preoperative antibody responses. The recurrence rate was assessed by the positivity of antibody responses at 3 and 12 months postoperatively.

**Results:**

Among 1001 patients, preoperative NY-ESO-1 and p53 antibody responses were positive in 12.6% and 18.1% of patients, respectively. NY-ESO-1 antibody responses became negative postoperatively in non-recurrent patients (negativity rates; 45% and 78% at 3 and 12 months, respectively), but remained positive in recurrent patients (negativity rates; 9% and 8%, respectively). p53 antibody responses remained positive in non-recurrent patients. In multivariate analysis, NY-ESO-1 antibody positivity at 3 months (*P*  < 0.03) and 12 months (*P*  < 0.001) were independent prognostic factors for a shorter recurrence-free interval.

**Conclusions:**

Serum NY-ESO-1 antibodies may be a useful predictive marker for postoperative recurrence in gastric cancer.

**Clinical trial registration:**

UMIN000007925.

## Introduction

Gastric cancer is the fourth leading cause of cancer-related death worldwide [1]. Advanced gastric cancer has a high recurrence rate even after curative surgery, leading to a poor prognosis overall. Early prediction of postoperative recurrence is important to determine the indication for intensive adjuvant chemotherapy and to improve treatment outcomes of advanced gastric cancer [2–4]. Although several methods have been developed to predict postoperative recurrence, no reliable predictive marker has been identified [5–9]. Carcinoembryonic antigen (CEA) and carbohydrate antigen 19-9 (CA19-9) are common tumor markers for gastric cancer in Japan, but these serum markers are positive in only 20–60% of cases and can predict recurrence approximately 3 months before image-based evidence of recurrence [10–12].

NY-ESO-1 antigen is one of the cancer-testis antigens initially identified by the serological analysis of recombinant cDNA expression libraries and is expressed in the normal testis, placenta, and tumor tissues [13]. They are highly tumor-specific and immunogenic and frequently induce specific immune responses [14, 15]. Spontaneous NY-ESO-1 antibody responses were observed in 9.7–11.1% of gastric cancers, 3.9–29.4% of esophageal cancers, 9.4% of melanomas, and 9–23% of lung cancers, but these responses were not detected in non-cancerous donors [16–19]. Therefore, NY-ESO-1 humoral immune responses may be used as a serological marker to detect these cancers. In our previous cohort study of gastric cancer, serum NY-ESO-1 antibody responses fell below the cut-off level after curative surgery in patients without recurrence and remained positive in patients with recurrence [20]. These findings suggest that postoperative persistent NY-ESO-1 antibody responses indicate the presence of minimal residual disease, and may predict postoperative recurrence.

The p53 tumor suppressor gene is a transcription factor that is mutated in over half of all human cancers [21]. These mutations generate an oncogenic mutated p53 protein that is a frequent driver mutation in various types of cancers [22]. Since overexpression of the p53 protein due to genetic alterations may stimulate anti-p53 immune responses, serum p53 antibody responses have been observed in various types of cancers, including 15–16% of gastric cancers, 32.9% of esophageal cancers, and 30% of colorectal cancers [16, 23, 24]. Currently, serum p53 antibodies are the only clinically available antibody marker [25].

Although postoperative persistent serum NY-ESO-1 antibody responses is a potential predictive marker of postoperative recurrence, its usefulness has not been fully investigated. Therefore, we designed a large multicenter observational study to examine the utility of serum NY-ESO-1 and p53 antibody responses as predictive markers of postoperative recurrence in gastric cancer. NY-ESO-1 and p53 humoral immune responses were analyzed serially over time in approximately 100 patients with recurrence after curative surgery.

## Materials and methods

### Study design and participants

This multicenter prospective observational study in 21 Japanese institutions assessed the utility of postoperative serum NY-ESO-1 and p53 antibody positivity to predict postoperative recurrence in gastric cancer. This study was organized by the Osaka University Clinical Research Group for Gastroenterological Study and was performed between January 1, 2012, and December 31, 2021. The inclusion criteria were newly diagnosed gastric cancer that could be curatively resected, histologically confirmed adenocarcinoma of the stomach, cT3–cT4b as per the 14th edition of the Japanese Classification of Gastric Carcinoma, no clinical distant metastasis (H0, P0, and M0), age 20–90 years, and Eastern Cooperative Oncology Group performance status of 0-2 [26]. Patients with distant metastasis were not included, but those with potentially resectable cStage IV disease after chemotherapy were eligible. Exclusion criteria were active synchronous cancer (synchronous coexisting cancer and metachronous cancer within 5 disease-free years), excluding carcinoma in situ. We planned to accumulate 100 patients with positive preoperative NY-ESO-1 antibody responses and postoperative recurrence. The total sample size was 1000 patients, because the recurrence rate and positivity of serum NY-ESO-1 antibody responses in cT3-4 gastric cancer were estimated to be 50% and 20%, respectively [2, 20].

### Management of postoperative follow-up

Patients underwent standard gastrectomy and lymph node dissection according to the Japanese Gastric Cancer Treatment Guidelines [27]. In brief, D2 lymphadenectomy was performed, but D1 plus lymphadenectomy was occasionally adopted for high-risk patients. The surgical approach and reconstruction method were not prespecified. The operative methods and pathology results were recorded according to the 14th edition of the Japanese Classification of Gastric Carcinoma [27]. Postsurgical management was performed according to the clinical protocol of each institution. Postoperative follow-up, including laboratory examinations and computed tomographic imaging, was performed every 3 months until 1 year, and thereafter every 6 months until 5 years after surgery. To ensure that patients followed up for fewer than 3 years were not evaluated as having no recurrence, recurrence was evaluated for at least 3 years after surgery, and patients followed up for fewer than 3 years without recurrence were excluded from the analysis. The median follow-up duration was 55.3 months (range; 0–129 months). Although adjuvant treatment was not prescribed, S-1 was administered in principle if R0 resection was performed and the pathological stage was II (excluding cT1) or III according to the Japanese Gastric Cancer Treatment Guidelines [27].

### Assessment of serum NY-ESO-1 and p53 antibodies

After primary enrollment, baseline serum NY-ESO-1 and p53 antibody responses were examined with blood samples. In patients whose NY-ESO-1 or p53 antibody responses were positive preoperatively, serum samples were serially collected every 3 months until 1 year, and thereafter every 6 months until 5 years after surgery. In patients with recurrence, serum samples were collected at the time of recurrence and thereafter every 3 months until either death or loss of follow-up. All serum samples were stored at −80 °C until analyzed.

### ELISA for serum NY-ESO-1 antibodies

Recombinant NY-ESO-1 protein (1 μM) in 100 µl of coating buffer (pH 9.6) was added onto a 96-well PolySorp immunoplate (Nunc, Roskilde, Denmark) and incubated overnight at 4 °C. Plates were washed with PBS and blocked with 5% FCS/PBS (200 μl/well) for 1 hour at room temperature. After washing, 100 μl of serially diluted serum was added to each well and incubated for 2 h at room temperature. After washing, horseradish peroxidase-conjugated goat anti-human IgG (MBL, Nagoya, Japan) was added to the wells, and the plates were incubated for 1 h at room temperature. After washing and development, absorbance was read at 490 nm. Levels of serum NY-ESO-1 antibody were assessed using optical density (O.D.) values. The cut-off value was defined as an O.D. value of 0.5 at a dilution of 1:100, which was the upper limit of the mean + 2× standard deviation values from 50 healthy donors [20].

### Detection of serum p53 antibodies, CEA and CA19-9

Serum p53 antibodies were detected by SRL, Inc. (Tokyo, Japan). A cut-off value of 1.3 U/ml was adopted to distinguish cancer patients from healthy persons [23]. Serum CEA and CA19-9 levels were measured by each hospital’s clinical laboratory department, and the cut-off values were 5.0 ng/ml and 37.0 U/ml, respectively.

### Endpoints

The primary endpoint was the percentage of patients with postoperative recurrence within 3 years after surgery based on the positivity of serum NY-ESO-1 or p53 antibody responses at 12 months after surgery in patients whose preoperative NY-ESO-1 or p53 antibody responses were positive. A post-hoc analysis was performed with the positivity of serum NY-ESO-1 or p53 antibody responses at 3 months after surgery. The secondary endpoints were the preoperative positivity of serum NY-ESO-1 and p53 antibody responses and the correlation between preoperative NY-ESO-1 or p53 antibody responses and prognosis.

### Statistics

Clinicopathological characteristics were compared using the *χ*^2^ test for categorical variables and the Mann–Whitney *U* test for continuous variables. For univariate analyses, the Kaplan–Meier estimator was used with the log-rank test. Recurrence-free interval (RFI) was measured from the date of surgery to the date of recurrence or death as a result of gastric cancer and was censored at the last follow-up or non-gastric-cancer-related death. Hazard ratios (HR) were estimated by univariate Cox proportional hazards regression models. In the multivariate analysis, a Cox proportional hazards regression model was fitted. All *P* values <0.05 were judged as statistically significant. Variables with *P* < 0.05 in univariate analysis were assessed in multivariate analysis. Statistical analyses were performed using the SPSS statistical package, version 22.0 (SPSS, Chicago, IL, USA).

## Results

### Patient population and backgrounds

**Fig. 1:**
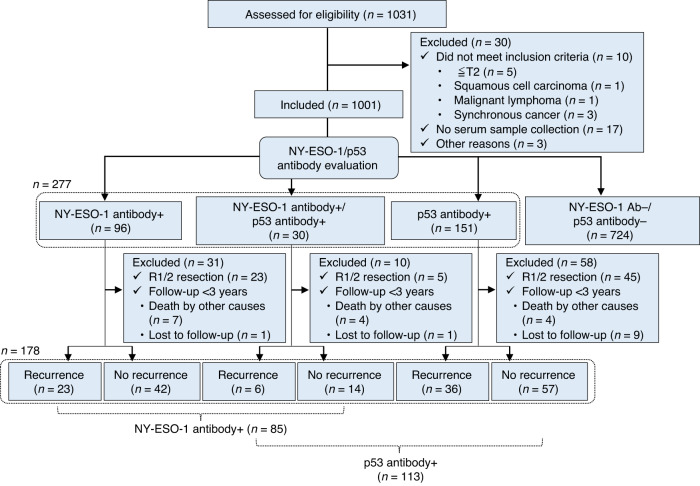
Schematic of the study design. The flowchart showes the subject enrollment, subsequent examinations and treatment outcome.

The study profile is illustrated in Fig. 1. Of 1031 patients enrolled from 21 institutions, 30 did not meet the inclusion criteria because of cT1-2 staging (*n* = 5), squamous cell carcinoma (*n* = 1), malignant lymphoma (*n* = 1), synchronous cancer (*n* = 3), no serum sample collection (*n* = 17), or other reasons (*n* = 3). In the remaining 1001 patients, baseline serum NY-ESO-1 and p53 antibody responses were positive in 126 (12.6%) and 181 (18.1%) patients, respectively. Of these 277 patients, 204 (73.6%) underwent R0 resection. Recurrence for 3 years could not be assessed in 26 patients due to death by other causes (*n* = 15) or other reasons (*n* = 11), so the full analysis comprised 178 patients with 3-year follow-up, of whom 85 and 113 were positive for NY-ESO-1 and p53 antibody responses, respectively. The baseline characteristics of the 1001 patients are shown in Table 1. All patients had cT3-4 disease, and 967 (96.7%) were cStage II–III. Among 944 dissected patients, 646 (68.4%) were pStage II–III. Positive NY-ESO-1 antibody responses were correlated with higher age (*P* = 0.012), male gender (*P* = 0.001), higher cN stage (*P* = 0.006), higher cStage (*P* = 0.026), and higher pT stage (*P* = 0.006). Positive p53 antibody response was not associated with any factors other than male gender (*P* = 0.022).

**Table 1:** Baseline characteristics of patients based on NY-ESO-1 and p53 antibody status.

	NY-ESO-1 antibody (−) (*n* = 875)	NY-ESO-1 antibody (+) (*n* = 126)	*P* value	p53 antibody (−) (*n* = 820)	p53 antibody (+) (*n* = 181)	*P* value
Age, years	72 (34–90)	74 (41–90)	0.012	72 (34–90)	73 (40–90)	0.59
Gender						
Male	599 (68.5%)	105 (83.3%)	0.001	564 (68.8%)	140 (73.3%)	0.022
Female	276 (31.5%)	21 (16.7%)		256 (31.2%)	41 (22.7%)	
cT						
3	430 (49.1%)	60 (47.6%)	0.75	406 (49.5%)	84 (46.4%)	0.45
4	445 (50.9%)	66 (52.4%)		414 (50.5%)	97 (53.6%)	
cN						
0	330 (37.7%)	28 (22.2%)	0.006	298 (36.3%)	60 (33.1%)	0.87
1	259 (29.6%)	43 (34.1%)		244 (29.8%)	58 (32.0%)	
2	210 (24.0%)	38 (30.2%)		202 (24.6%)	46 (25.4%)	
3	76 (8.7%)	17 (13.5%)		76 (9.3%)	17 (9.4%)	
cStage						
II	439 (50.2%)	47 (37.3%)	0.026	401 (48.9%)	85 (47.0%)	0.853
III	407 (46.5%)	74 (58.7%)		392 (47.8%)	89 (49.2%)	
IV	29 (3.3%)	5 (4.0%)		27 (3.3%)	7 (3.9%)	
Tumor location						
Lower	341 (39.0%)	42 (36.5%)	0.058	312 (38.0%)	71 (39.2%)	0.71
Middle	322 (36.8%)	41 (33.3%)		302 (36.8%)	61 (33.7%)	
Upper	212 (24.2%)	43 (30.2%)		206 (25.1%)	49(27.1%)	
Histological type^a^						
Differentiated	396 (45.3%)	73 (57.9%)	0.068	378 (46.1%)	91 (50.3%)	0.60
Undifferentiated	383 (43.8%)	42 (33.3%)		356 (43.4%)	69 (38.1%)	
Others	45 (5.1%)	5 (4.0%)		41 (5.0%)	9 (5.0%)	
No dissection	51 (5.8%)	6 (4.8%)		45 (5.5%)	12 (6.6%)	
Neoadjuvant chemotherapy						
No	783 (89.5%)	115 (91.3%)	0.54	738 (90.0%)	160 (88.4%)	0.52
Yes	92 (10.5%)	11 (8.7%)		82 (10.0%)	21 (11.6%)	
Surgical curability						
R0	680 (77.7%)	98 (77.8%)	0.74	647 (78.9%)	131 (72.4%)	0.24
R1	95 (10.9%)	14 (11.1%)		83 (10.1%)	26 (14.4%)	
R2	98 (11.2%)	13 (10.3%)		88 (10.7%)	23 (12.7%)	
No operation	2 (0.2%)	1 (0.8%)		2 (0.2%)	1 (0.6%)	
pT^b^						
0	6 (0.7%)	1 (0.8%)	0.006	7 (0.9%)	0 (0%)	0.31
1	82 (10.0%)	6 (5.0%)		77 (9.9%)	11 (6.5%)	
2	93 (11.3%)	25 (20.8%)		92 (11.9%)	26 (15.4%)	
3	299 (36.3%)	51 (42.5%)		285 (36.8%)	65 (38.5%)	
4	344 (41.7%)	37 (30.8%)		314 (40.5%)	67 (39.6%)	
pN^b^						
0	267 (32.4%)	28 (23.3%)	0.065	243 (31.4%)	52 (30.8%)	0.80
1	150 (18.2%)	25 (20.8%)		146 (18.8%)	29 (17.2%)	
2	160 (19.4%)	19 (15.8%)		149 (19.2%)	30 (17.8%)	
3	247 (30.0%)	48 (40.0%)		237 (30.6%)	58 (34.3%)	
pStage^b^						
0	6 (0.7%)	1 (0.8%)	0.82	7 (0.9%)	0 (0%)	0.15
I	124 (15.0%)	13 (10.8%)		116 (15.0%)	21 (12.4%)	
II	242 (29.4%)	37 (30.8%)		225 (29.0%)	54 (32.0%)	
III	319 (38.7%)	48 (40.0%)		309 (39.9%)	58 (34.3%)	
IV	133 (16.1%)	21 (17.5%)		118 (15.2)	36 (21.3%)	
Adjuvant chemotherapy						
No	783 (89.5%)	115 (91.3%)	0.54	738 (90.0%)	160 (88.4%)	0.52
Yes	92 (10.5%)	11 (8.7%)		82 (10.0%)	21 (11.6%)	

Data are shown as the number of patients (%) or the median (min–max). The TNM stage was classified according to the 14th edition of the Japanese Classification of Gastric Carcinoma.^26^

^a^Differentiated: pap/tub1/tub2, Undifferentiated: por/sig, Others: muc/NEC/carcinoma with lymphoid stroma/hepatoid adenocarcinoma/undifferentiated carcinoma.

^b^Pathological data are shown only for dissected patients.

### Prediction of recurrence by NY-ESO-1 antibody responses

**Fig. 2:**
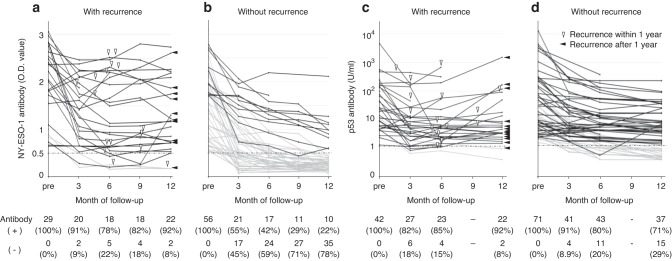
Longitudinal changes in serological immune responses to NY-ESO-1 and p53 in patients with positive preoperative antibody responses. NY-ESO-1 antibody responses in 29 patients with recurrence (**a**) and in 56 patients without recurrence (**b**). The cut-off value of NY-ESO-1 antibody responses was an O.D. value of 0.5 at a serum dilution of 1:100. p53 antibody responses in 42 patients with recurrence (**c**) and in 71 patients without recurrence (**d**). The cut-off value of p53 antibody responses was 1.3 U/ml. Black lines indicate the data of patients whose antibody values did not fall below the cut-off level at 12 months, and gray lines indicate the data of patients whose antibody values fell below the cut-off level at 12 months. White and black wedges indicate the timing of recurrence within 1 year and after 1 year, respectively.

**Fig. 3:**
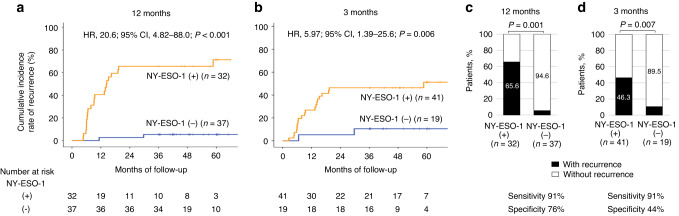
Recurrence rates based on the postoperative evaluation of serum NY-ESO-1 antibody responses. Kaplan–Meier analysis of cumulative recurrence rates was assessed based on the NY-ESO-1 antibody status at 12 months (**a**) and 3 months (**b**) after surgery. HRs and 95% CIs were calculated using the Cox proportional hazard model. *P* values were calculated using the two-sided log-rank test. Percentages of patients with or without recurrence based on NY-ESO-1 antibody status at 12 months (**c**) and 3 months (**d**) after surgery. *P* values were calculated using a two-sided chi-squared test comparing the distribution of factors between the two columns (positive vs negative). The sensitivity and specificity for postoperative recurrence assessed by NY-ESO-1 positivity are shown below the graph.

Among those who underwent radical resection, there was no difference in the recurrence rate according to the preoperative status of NY-ESO-1 antibody responses (Supplementary Fig. 1a). Recurrence occurred in 29 of the 85 patients with positive preoperative NY-ESO-1 antibody responses, who underwent R0 resection and had sufficient follow-up periods (Fig. 1). Among patients with recurrence, serum NY-ESO-1 antibody responses became negative in 2 (9%) and 2 (8%) patients at 3 and 12 months, respectively (Fig. 2a), compared to 17 (45%) and 35 (78%) patients at 3 and 12 months among patients without recurrence, respectively (Fig. 2b). Positive NY-ESO-1 antibody responses at 12 months were correlated with higher pN stage and pStage (Supplementary Table 1). The cumulative recurrence rate was significantly higher in patients with positive NY-ESO-1 antibody responses at 12 months (HR, 20.6; 95% CI, 4.82–88.0; *P*  < 0.001) (Fig. 3a) and 3 months (HR, 5.97; 95% CI, 1.39–25.6; *P*  < 0.006) (Fig. 3b) than in patients with negative responses. The sensitivity and specificity for postoperative recurrence within 3 years were 91% and 76% for serum NY-ESO-1 antibody responses at 12 months, respectively (Fig. 3c), and 91% and 44% for those at 3 months, respectively (Fig. 3d).

### Prediction of recurrence by p53 antibody responses

**Fig. 4:**
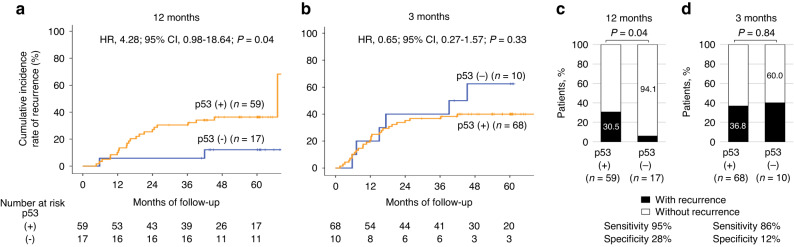
Recurrence rates based on the postoperative evaluation of serum p53 antibody responses. Kaplan–Meier analysis of cumulative recurrence rates was assessed based on the p53 antibody status at 12 months (**a**) and 3 months (**b**) after surgery. HRs and 95% CIs were calculated using the Cox proportional hazard model. *P* values were calculated using the two-sided log-rank test. Percentages of patients with or without recurrence based on p53 antibody status at 12 months (**c**) and 3 months (**d**) after surgery. *P* values were calculated using a two-sided chi-squared test comparing the distribution of factors between the two columns (positive vs negative). The sensitivity and specificity for postoperative recurrence assessed by p53 positivity are shown below the graph.

Among those who underwent radical resection, there was no difference in the recurrence rate according to the preoperative status of p53 antibody responses (Supplementary Fig. 1b). Recurrence occurred in 42 of the 113 patients with positive preoperative p53 antibody responses, who underwent R0 resection and had sufficient follow-up periods (Fig. 1). Among patients with recurrence, serum p53 antibody responses became negative in 6 (18%) and 2 (8%) patients at 3 and 12 months, respectively (Fig. 2c), compared to 4 (9%) and 15 (29%) of patients at 3 and 12 months among patients without recurrence, respectively (Fig. 2d). Positive p53 antibody responses at 12 months were not associated with any background factors (Supplementary Table 2). The cumulative recurrence rate was significantly higher in patients with positive p53 antibody responses at 12 months than in those with negative responses (HR, 4.28; 95% CI, 0.98–18.5; *P*  = 0.04) (Fig. 4a), but there was no significant difference between patients with positive p53 antibody responses at 3 months and negative responses (HR, 0.65; 95% CI, 0.27–1.57; *P* = 0.33) (Fig. 4b). The sensitivity and specificity for postoperative recurrence within 3 years were 95% and 28% for serum p53 antibody responses at 12 months (Fig. 4c), and 86% and 12% for those at 3 months, respectively (Fig. 4d).

### Prediction of recurrence by CEA and CA19-9

A similar analysis involving serum CEA and CA19-9 levels was performed in the same 178 patients with positive preoperative serum NY-ESO-1 or p53 antibody responses. Serum levels of both CEA and CA19-9 quickly fell below the cut-off for positivity after curative surgery regardless of recurrence and rose above the cut-off after the onset of recurrence (Supplementary Figs. 2 and 3). The sensitivity and specificity for postoperative recurrence within 3 years were 87% and 44% for CEA levels at 12 months, 42% and 71% for CEA levels at 3 months, 56% and 67% for CA19-9 levels at 12 months, and 22% and 67% for CA19-9 at 3 months, respectively (Supplementary Fig. 4).

### Prognostic analysis for recurrence by multiple factors

Univariate and multivariate analyses of RFI were conducted in patients with positive preoperative NY-ESO-1 antibody responses. In a univariate analysis of 69 patients whose serum NY-ESO-1 antibody responses were evaluated at 12 months, total gastrectomy, pT3-4, pN2-3, pStage III–IV, and a positive NY-ESO-1 antibody response at 12 months were poor prognostic factors (Table 2). In a multivariate analysis, a positive NY-ESO-1 antibody response at 12 months (HR, 21.6; 95% CI, 4.75-98.7; *P*  < 0.001), total gastrectomy, and pT3-4 were independent poor prognostic factors associated with RFI. Multivariate analysis of 60 patients whose serum NY-ESO-1 antibody responses were evaluated at 3 months also showed that a positive NY-ESO-1 antibody response at 3 months (HR, 5.31; 95% CI, 1.19–23.7; *P*  = 0.03) was an independent poor prognostic factor correlated with RFI (Supplementary Table 3). However, a positive p53 antibody response at 12 months was not an independent prognostic factor with RFI in a multivariate analysis of 76 patients whose serum p53 antibody responses were evaluated at 12 months (Supplementary Table 4). Also, a positive p53 antibody response at 3 months was not an independent prognostic factor with RFI in a multivariate analysis of 78 patients whose serum p53 antibody responses were evaluated at 3 months (Supplementary Table 5).

**Table 2:** Univariate and multivariate analyses of recurrence-free interval in 69 patients whose NY-ESO-1 antibody responses were evaluated at 12 months.

	Univariate analysis HR (95% CI)	*P* value	Multivariate analysis HR (95% CI)	*P* value
Age (years) (≥70 vs. <70)	1.08 (0.48–2.44)	0.85		
Sex (female vs. male)	0.79 (0.23–2.64)	0.70		
Tumor location (lower, middle vs. upper)	0.93 (0.85–1.02)	0.12		
Histology (differentiated vs. other)	0.93 (0.39–2.24)	0.87		
Neoadjuvant chemotherapy (yes vs. no)	1.48 (0.44–4.97)	0.52		
Procedure (total vs. partial gastrectomy)	2.57 (1.12–5.88)	0.02	4.38 (1.77–10.9)	0.001
pT (3, 4 vs. 0, 1, 2)	1.07 (1.01–1.13)	0.007	1.09 (1.03–1.15)	0.004
pN (2, 3 vs. 0, 1)	1.15 (1.06–1.25)	<0.001	1.08 (0.99–1.17)	0.09
pStage (III, IV vs. 0, I, II)	1.02 (1.01–1.04)	<0.001		
NY-ESO-1 antibody status at 12 months (positive vs. negative)	20.6 (4.82–88.0)	<0.001	21.6 (4.75–98.7)	<0.001

*HR* hazard ratio, *CI* confidence interval.

Variables with *P* < 0.05 in univariate analysis were assessed in multivariate analysis.

## Discussion

This large multicenter observational study proved that positive serum NY-ESO-1 antibody responses at 3 and 12 months after surgery predicted postoperative recurrence in gastric cancer patients whose antibody responses were positive preoperatively. In contrast, serum p53 antibody responses did not accurately predict postoperative recurrence in patients with positive preoperative p53 antibody responses. The fact that preoperative serum NY-ESO-1 antibody status was not correlated with recurrence, but persistent positive postoperative NY-ESO-1 antibody responses were significantly associated with higher recurrence rate, suggests that postoperative assessment of NY-ESO-1 antibody responses, rather than preoperative, is important in predicting recurrence. This means that the continued detection of postoperative NY-ESO-1 antibody responses may reflect the presence of minimal residual disease following surgery. Given that there is currently no marker that predicts recurrence soon after radical resection of gastric cancer, early postoperative evaluation of serum NY-ESO-1 antibody responses to predict recurrence is considered very meaningful, including its impact on the therapeutic strategy of postoperative treatment.

Serum CEA and CA19-9 are common tumor markers for gastric cancer, but their predictive values for postoperative recurrence are not very high [10]. In our analysis of patients whose preoperative serum CEA and CA19-9 were positive, the values of these markers decreased in the early postoperative period and then increased after the onset of recurrence. The results indicated the difficulty of predicting postoperative recurrence with CEA or CA19-9 early after curative surgery. Circulatory tumor DNA (ctDNA), which leaks into the bloodstream from tumor cells and may reflect minimal residual disease, has been intensively studied as a biomarker of recurrence in various types of cancers [28–37]. In colorectal cancer, ctDNA was reported to reflect disease activity and predict recurrence during the early postoperative period [28, 29]. The evaluation of ctDNA may be useful to determine the indication for adjuvant chemotherapy, potentially impacting postoperative management [31, 35, 37]. The detection rate has been increasing, ranging from 78.0 to 99.1% preoperatively, depending on the specific target genes, cancer types, and detection methods [28–37]. However, ctDNA analysis requires whole-exome sequencing, which is expensive and takes a couple of weeks. On the other hand, serum NY-ESO-1 antibody responses can be evaluated with a small amount of serum and a one-day ELISA assay. Thus, this method of evaluating serum NY-ESO-1 antibodies is much faster and less expensive than ctDNA analysis. Given the convenience of the detection method as well as its ability to predict recurrence, postoperative evaluation of serum NY-ESO-1 antibody responses would be a powerful tool to predict postoperative recurrence in the early postoperative period, thereby allowing an appropriate selection of patients for intensive adjuvant therapy. Moreover, considering the relatively high rates of spontaneous NY-ESO-1 antibody responses in other types of cancers (3.9–29.4% of esophageal cancers, 9.4% of melanomas, and 9–23% of lung cancers) [16–19], postoperative recurrence can be predicted also in other malignancies with positive NY-ESO-1 antibody responses, which may be a major advantage of performing this analysis.

The results of this study suggest that NY-ESO-1 antibody response is a predictive marker for postoperative recurrence, but p53 antibodies is not. The discrepancy between NY-ESO-1 and p53 may be partly caused by the difference in the half-life of the antibody response to each antigen. In the recent reports on antibody responses to covid-19, antibody responses gradually decreased during 6 months after covid-19 vaccination [38, 39]. In contrast, the antibody responses to the hepatitis B virus remained high for more than 10 years after vaccination [40]. The half-life of the antibody responses varies greatly from antigen to antigen, and the half-life of p53 antibody may be longer than that of NY-ESO-1 antibody. Another plausible explanation is the difference in the immune response to mutated and unmutated antigens. Since tumors produce various mutated p53 proteins, p53 antibodies may react to both the mutated and unmutated proteins [41, 42]. On the other hand, the NY-ESO-1 antigen is an unmutated protein present only in the testis and tumor. This difference between mutated and unmutated antigens may account for the difference in the strength of immune response between NY-ESO-1 and p53. Furthermore, it is possible that adjuvant chemotherapy had different effects on the antibody responses to NY-ESO-1 and p53. This issue has not been well studied, and should be examined in the future.

In recent years, immune checkpoint inhibitors (ICIs) have been administered for a variety of cancer types. The presence of anti-tumor antigen-specific T cells is considered essential for the therapeutic effect of ICIs [43]. Mutation-derived antigens, so-called neoantigens, have been intensively studied in recent years among tumor antigens, and anti-neoantigen-specific T cells were reported to increase in number during ICI treatment in responders [44, 45]. Similarly, it is possible that anti-cancer testis antigen-specific T cells attack tumors expressing cancer-testis antigens in ICI treatment. Indeed, a report showed that melanoma with positive NY-ESO-1 immune response responded to anti–CTLA-4 therapy [46], and other reports showed that lung adenocarcinoma with serum NY-ESO-1 antibody responses had a better clinical efficacy to anti-PD-1 therapy than those without antibody responses [47, 48]. Thus, gastric cancer with persistent NY-ESO-1 antibody responses after surgery are prone to recurrence, but may respond to ICI treatment, suggesting that the use of ICI as postoperative adjuvant therapy may improve outcome. Postoperative evaluation of serum NY-ESO-1 antibodies may be important not only for predicting postoperative recurrence but also for implementing postoperative precision medicine including immunotherapy. Moreover, the use of adjuvant chemotherapy had no particular impact on prognosis, in every subgroup categorized by postoperative NY-ESO-1 or p53 status at 3 and 12 months. (Supplementary Figs. 5 and 6). Since adjuvant chemotherapy was more frequently used in patients with higher postoperative stage, which could affect the results in this study, further studies will be needed to investigate whether adjuvant chemotherapy can improve the survival outcome in patients with positive postoperative NY-ESO-1 antibody responses or can be omitted in patients with negative conversion of NY-ESO-1 antibody responses.

This study had several limitations. First, the evaluation of serum NY-ESO-1 antibody responses was based on O.D. values by ELISA, and specific concentrations was not measured. Regarding this point, we have developed a rapid immunoassay system to specifically and quantitatively measure serum NY-ESO-1 antibody levels in clinical practice [48]. Serum NY-ESO-1 antibody levels measured with ELISA and the rapid immunoassay system were very similar, and both assays used the same NY-ESO-1 antigen. Thus, quantification will be possible in the future. Second, serum NY-ESO-1 antibody responses were not evaluated before 3 months. Since the earlier prediction of postoperative recurrence is more meaningful, examining whether the measurement of NY-ESO-1 antibody responses at earlier time points will be necessary to predict postoperative recurrence.

In conclusion, this large multicenter observational study showed that positive serum NY-ESO-1 antibody responses after curative surgery predicted the postoperative recurrence of gastric cancer patients whose antibody responses were positive preoperatively. Serum NY-ESO-1 antibody evaluation may be used to determine the indication of intensive adjuvant therapy after curative surgery in patients with NY-ESO-1-expressing malignancies.

### Supplementary information

Supplementary Table and Figure

REMARK checklist

## Data Availability

The datasets generated and/or analyzed during the current study are available from the corresponding author on reasonable request.
