# Supplementary material for: Serum NY-ESO-1 antibody as a predictive biomarker for postoperative recurrence of gastric cancer: a multicenter prospective observational study
Source: Br J Cancer. 2024 Feb 7;130(7):1157–65. doi: 10.1038/s41416-023-02540-3 (PMC10991393; doi:10.1038/s41416-023-02540-3)
Supplement: Supplementary file 1 — Supplementary Table and Figure [file 41416_2023_2540_MOESM1_ESM.pdf]

Supplementary Table 1. Patient characteristics based on serum NY-ESO-1 antibody status at 12 months after surgery.

		NY-ESO-1 Antibody (-) (n=37)	NY-ESO-1 Antibody (+) (n=32)	*P* value
Age	years (range)	73 (41-86)	72.5 (61-82)	0.49
Sex	Male	32 (86.5%)	27 (84.4%)	0.80
	Female	5 (13.5%)	5 (15.6%)	
cT	3	19 (51.4%)	20 (62.5%)	0.35
	4	18 (48.6%)	12 (37.5%)	
cN	0	9 (24.3%)	3 (9.4%)	0.26
	1	13 (35.1%)	15 (46.9%)	
	2	9 (24.3%)	11 (34.4%)	
	3	6 (16.2%)	3 (9.4%)	
cStage	II	18 (48.6%)	10 (31.3%)	0.22
	III	19 (51.4%)	21 (65.6%)	
	IV	0 (0%)	1 (3.1%)	
Tumor location	Lower	13 (35.1%)	5 (15.6%)	0.18
	Middle	10 (27.0%)	12 (37.5%)	
	Upper	14 (37.8%)	15 (46.9%)	
Histological type [a]	Differentiated	25 (67.6%)	24 (75.0%)	0.39
	Undifferentiated	10 (27.0%)	8 (25.0%)	
	Others	2 (5.4%)	0 (0%)	
Neoadjuvant chemotherapy	Yes	2 (5.4%)	5 (15.6%)	0.16
	No	35 (94.6%)	27 (84.4%)	
Surgical procedure	Total gastrectomy	15 (40.5%)	16 (50.0%)	0.43
	Partial gastrectomy	22 (59.5%)	16 (50.0%)	
pT [b]	0	0 (0%)	1 (3.1%)	0.37
	1	4 (10.8%)	1 (3.1%)	
	2	11 (29.7%)	6 (18.8%)	
	3	14 (37.8%)	17 (53.1%)	
	4	8 (21.6%)	7 (21.9%)	
pN [b]	0	12 (32.4%)	2 (6.3%)	0.001
	1	14 (37.8%)	5 (15.6%)	
	2	4 (10.8%)	6 (18.8%)	
	3	7 (18.9%)	19 (59.4%)	
pStage [b]	0	0 (0%)	1 (3.1%)	0.003
	I	8 (21.6%)	0 (0%)	
	II	17 (45.9%)	9 (28.1%)	
	III	11 (29.7%)	22 (68.8%)	
	IV	1 (2.7%)	0 (0%)	
Adjuvant chemotherapy	No	15 (40.5%)	6 (18.8%)	0.050
	Yes	22 (59.5%)	26 (81.3%)	

Data are shown as the number of patients (%) or the median (min-max). The TNM stage was classified according to the 14th edition of the Japanese Classification of Gastric Carcinoma.[26] [a] Differentiated: pap/tub1/tub2, Undifferentiated: por/sig, Others: muc/NEC/carcinoma with lymphoid stroma/hepatoid adenocarcinoma/undifferentiated carcinoma. [b] Pathological data are shown only for dissected patients.

Supplementary Table 2. Patient characteristics based on serum p53 antibody status at 12 months after surgery.

		p53 Antibody (-) (n=17)	p53 Antibody (+) (n=59)	*P* value
Age	years (range)	73 (49-84)	68 (40-88)	0.16
Sex	Male	13 (76.5%)	47 (79.7%)	0.78
	Female	4 (23.5%)	12 (20.3%)	
cT	3	7 (41.2%)	33 (55.9%)	0.28
	4	10 (58.8%)	26 (44.1%)	
cN	0	4 (23.5%)	26 (44.1%)	0.36
	1	5 (29.4%)	17 (28.8%)	
	2	6 (35.3%)	11 (18.6%)	
	3	2 (11.8%)	5 (8.5%)	
cStage	II	7 (41.2%)	35 (59.3%)	0.36
	III	9 (52.9%)	20 (33.9%)	
	IV	1 (5.9%)	4 (6.8%)	
Tumor location	Lower	8 (47.1%)	20 (33.9%)	0.61
	Middle	5 (29.4%)	22 (37.3%)	
	Upper	4 (23.5%)	17 (28.8%)	
Histological type [a]	Differentiated	9 (52.9%)	32 (54.2%)	0.16
	Undifferentiated	6 (35.3%)	26 (44.1%)	
	Others	2 (11.8%)	1 (1.7%)	
Neoadjuvant chemotherapy	Yes	3 (17.6%)	11 (18.6%)	0.93
	No	14 (82.4%)	48 (81.4%)	
Surgical procedure	Total gastrectomy	5 (29.4%)	20 (33.9%)	0.73
	Partial gastrectomy	12 (70.6%)	39 (66.1%)	
pT [b]	1	2 (11.8%)	4 (6.8%)	0.79
	2	4 (23.5%)	15 (25.4%)	
	3	8 (47.1%)	24 (40.7%)	
	4	3 (17.6%)	16 (27.1%)	
pN [b]	0	6 (35.3%)	23 (39.0%)	0.31
	1	6 (35.3%)	9 (15.3%)	
	2	2 (11.8%)	12 (20.3%)	
	3	3 (17.6%)	15 (25.4%)	
pStage [b]	I	4 (23.5%)	8 (13.6%)	0.17
	II	7 (41.2%)	25 (42.4%)	
	III	5 (29.4%)	26 (44.1%)	
	IV	1 (5.9%)	0 (0%)	
Adjuvant chemotherapy	No	10 (58.8%)	15 (25.4%)	0.010
	Yes	7 (41.2%)	44 (74.6%)	

Data are shown as the number of patients (%) or the median (min-max). The TNM stage was classified according to the 14th edition of the Japanese Classification of Gastric Carcinoma.[26] [a] Differentiated: pap/tub1/tub2, Undifferentiated: por/sig, Others: muc/NEC/carcinoma with lymphoid stroma/hepatoid adenocarcinoma/undifferentiated carcinoma. [b] Pathological data are shown only for dissected patients.

Supplementary Table 3. Univariate and multivariate analyses of recurrence-free interval in 60 patients whose NY-ESO-1 antibody responses were evaluated at 3 months

	Univariate analysis HR (95% CI)	*P* value	Multivariate analysis HR (95% CI)	*P* value
Age (Years) (≥70 vs. <70)	1.12 (0.48-2.61)	0.80		
Sex (Female vs. Male)	0.73 (0.22-2.46)	0.61		
Tumor location (Lower, Middle vs. Upper)	0.89 (0.81-0.98)	0.02	0.96 (0.85-1.09)	0.54
Histology (Differentiated vs. Other)	0.82 (0.33-2.00)	0.66		
Neoadjuvant chemotherapy (Yes vs. No)	1.37 (0.40-4.62)	0.61		
Procedure (Total vs. Partial gastrectomy)	2.99 (1.25-7.15)	0.01	2.77 (0.85-9.08)	0.09
pT (3, 4 vs. 0, 1, 2)	1.07 (1.01-1.13)	0.01	1.07 (1.01-1.13)	0.03
pN (2, 3 vs. 0, 1)	1.12 (1.03-1.22)	0.003	1.08 (0.99-1.18)	0.07
pStage (III, IV vs. 0, I, II)	1.02 (1.01-1.03)	0.001		
NY-ESO-1 antibody status at 3 months (Positive vs. Negative)	5.97 (1.39-25.6)	0.006	5.31 (1.19-23.7)	0.03

HR, hazard ratio; CI, confidence interval
Variables with $p<0.05$ in univariate analysis were assessed in multivariate analysis.

Supplementary Table 4. Univariate and multivariate analyses of recurrence-free interval in 76 patients whose p53 antibody responses were evaluated at 12 months

	Univariate analysis HR (95% CI)	*P* value	Multivariate analysis HR (95% CI)	*P* value
Age (Years) (≥70 vs. <70)	0.37 (0.16-0.91)	0.02	0.44 (0.18-1.07)	0.07
Sex (Female vs. Male)	0.73 (0.25-2.15)	0.57		
Tumor location (Lower, Middle vs. Upper)	0.94 (0.86-1.03)	0.17		
Histology (Differentiated vs. Other)	0.85 (0.38-1.89)	0.68		
Neoadjuvant chemotherapy (Yes vs. No)	1.82 (0.72-4.62)	0.20		
Procedure (Total vs. Partial gastrectomy)	2.35 (1.06-5.24)	0.03	2.40 (1.06-5.43)	0.04
pT (3, 4 vs. 0, 1, 2)	1.02 (0.98-1.07)	0.27		
pN (2, 3 vs. 0, 1)	1.03 (0.97-1.10)	0.30		
pStage (III, IV vs. 0, I, II)	1.01 (1.00-1.02)	0.04	1.01 (1.00-1.02)	0.17
p53 antibody status at 12 months (Positive vs. Negative)	4.28 (0.98-18.6)	0.04	4.27 (0.96-18.9)	0.06

HR, hazard ratio; CI, confidence interval
Variables with $p<0.05$ in univariate analysis were assessed in multivariate analysis.

Supplementary Table 5. Univariate and multivariate analyses of recurrence-free interval in 78 patients whose p53 antibody responses were evaluated at 3 months

	Univariate analysis HR (95% CI)	*P* value	Multivariate analysis HR (95% CI)	*P* value
Age (Years) (≥70 vs. <70)	0.59 (0.29-1.18)	0.13		
Sex (Female vs. Male)	0.52 (0.20-1.34)	0.17		
Tumor location (Lower, Middle vs. Upper)	0.94 (0.87-1.02)	0.10		
Histology (Differentiated vs. Other)	0.96 (0.48-1.91)	0.90		
Neoadjuvant chemotherapy (Yes vs. No)	1.66 (0.75-3.70)	0.21		
Procedure (Total vs. Partial gastrectomy)	2.05 (1.03-4.09)	0.04	1.84 (0.92-3.71)	0.09
pT (3, 4 vs. 0, 1, 2)	1.03 (0.99-1.07)	0.14		
pN (2, 3 vs. 0, 1)	1.04 (0.99-1.09)	0.16		
pStage (III, IV vs. 0, I, II)	1.01 (1.00-1.02)	0.02	1.01 (1.00-1.02)	0.049
p53 antibody status at 12 months (Positive vs. Negative)	0.65 (0.27-1.57)	0.33		

HR, hazard ratio; CI, confidence interval
Variables with $p<0.05$ in univariate analysis were assessed in multivariate analysis.

Supplementary Figure 1.

Recurrence rates based on the preoperative evaluation of serum NY-ESO-1 or p53 antibody responses in patients with curative resection.

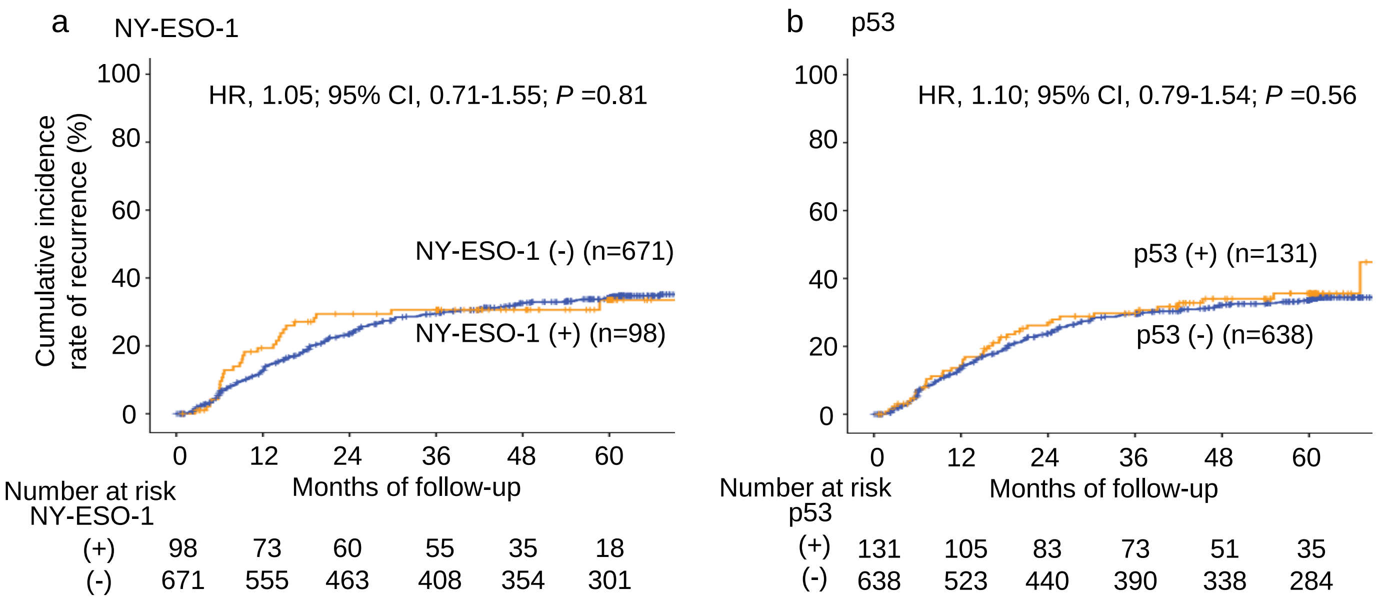

Kaplan-Meier analysis of cumulative recurrence rates was assessed based on the preoperative NY-ESO-1 and p53 antibody status (a and b). HRs and 95% CIs were calculated using the Cox proportional hazard model. P values were calculated using the two-sided log-rank test.

Supplementary Figure 2.

Longitudinal changes in serum CEA levels in patients with positive preoperative CEA.

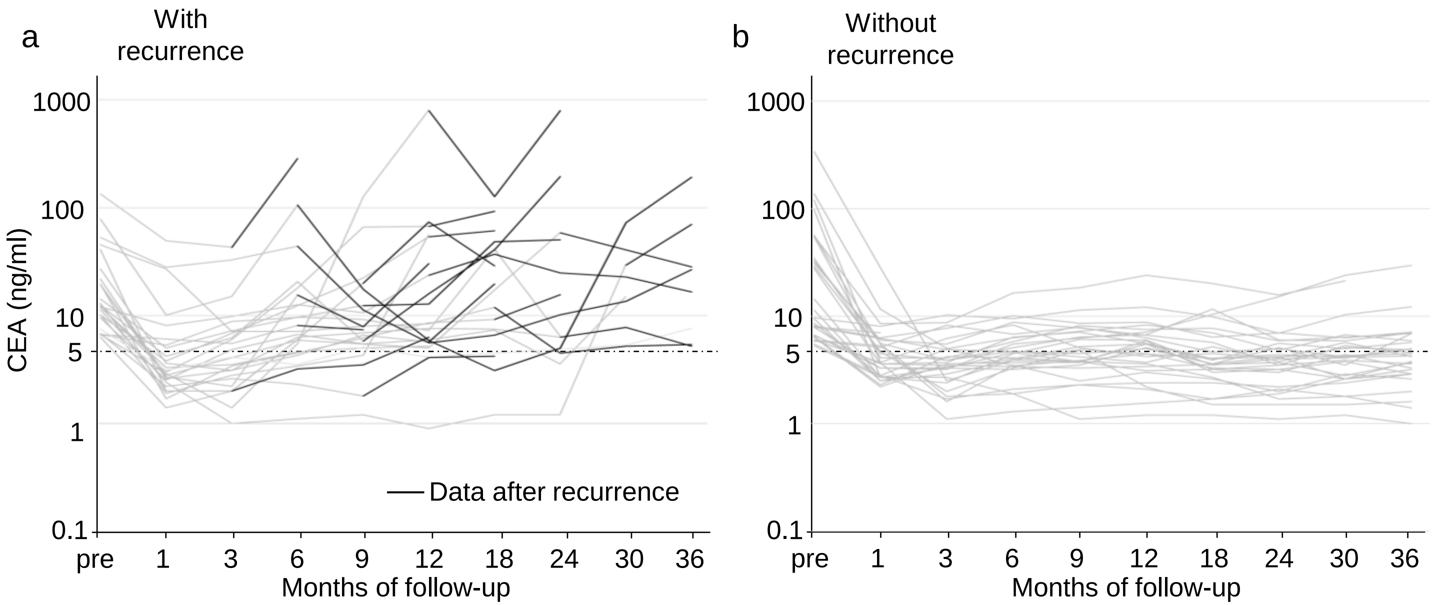

CEA	pre	1	3	6	9	12
(+)	26 (100%)	7 (28%)	10 (42%)	17 (68%)	19 (83%)	20 (87%)
(-)	0 (0%)	18 (72%)	14 (58%)	8 (32%)	4 (17%)	3 (13%)

CEA	pre	1	3	6	9	12
(+)	30 (100%)	12 (40%)	8 (29%)	11 (39%)	11 (46%)	15 (56%)
(-)	0 (0%)	18 (60%)	20 (71%)	17 (61%)	13 (54%)	12 (44%)

(a) Patients with recurrence (n=26) and (b) patients without recurrence (n=38). Gray and black lines indicate the data before and after recurrence, respectively. The cut-off value of CEA was 5 ng/ml.

Supplementary Figure 3.

Longitudinal changes in serum CA19-9 levels in patients with positive preoperative CA19-9.

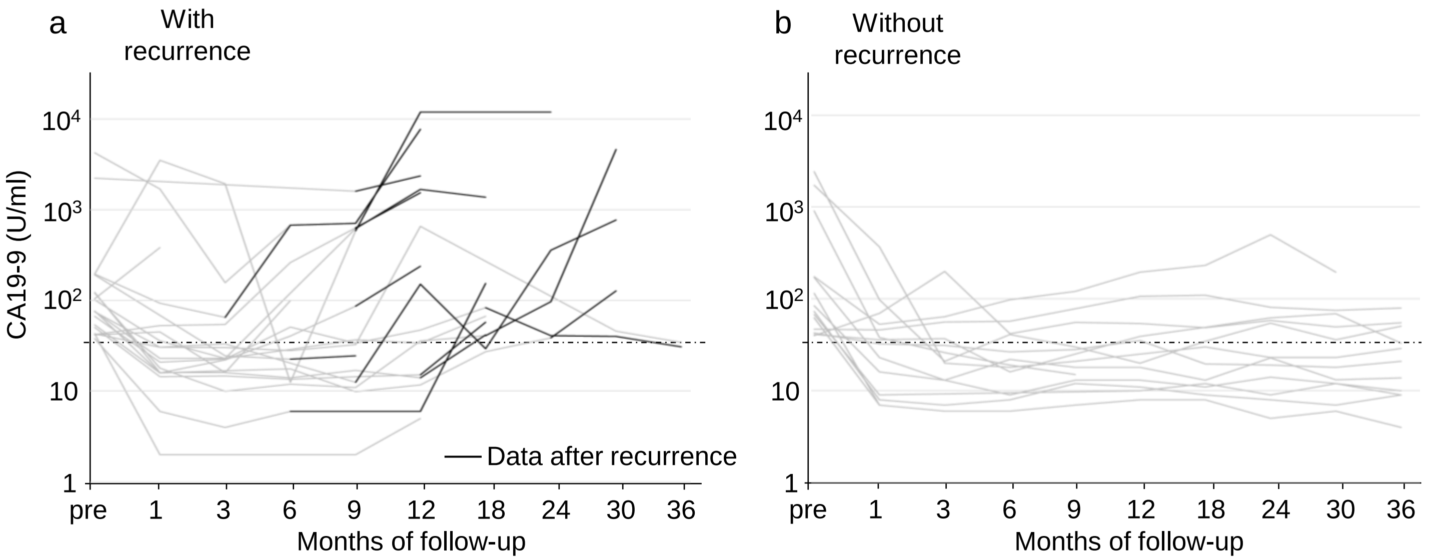

CA19-9	pre	1	3	6	9	12
a (+)	20 (100%)	6 (33%)	4 (22%)	7 (41%)	7 (41%)	9 (56%)
a (-)	0 (0%)	12 (67%)	14 (78%)	10 (59%)	10 (59%)	7 (44%)
b (+)	14 (100%)	6 (43%)	4 (33%)	4 (33%)	2 (20%)	4 (33%)
b (-)	0 (0%)	8 (57%)	8 (67%)	8 (67%)	8 (80%)	8 (67%)

(a) Patients with recurrence (n=20) and (b) patients without recurrence (n=19). Gray and black lines indicate the data before and after recurrence, respectively. The cut-off value of CA19-9 was 37 U/ml.

Supplementary Figure 4.

Recurrence rates based on the postoperative evaluation of CEA and CA19-9.

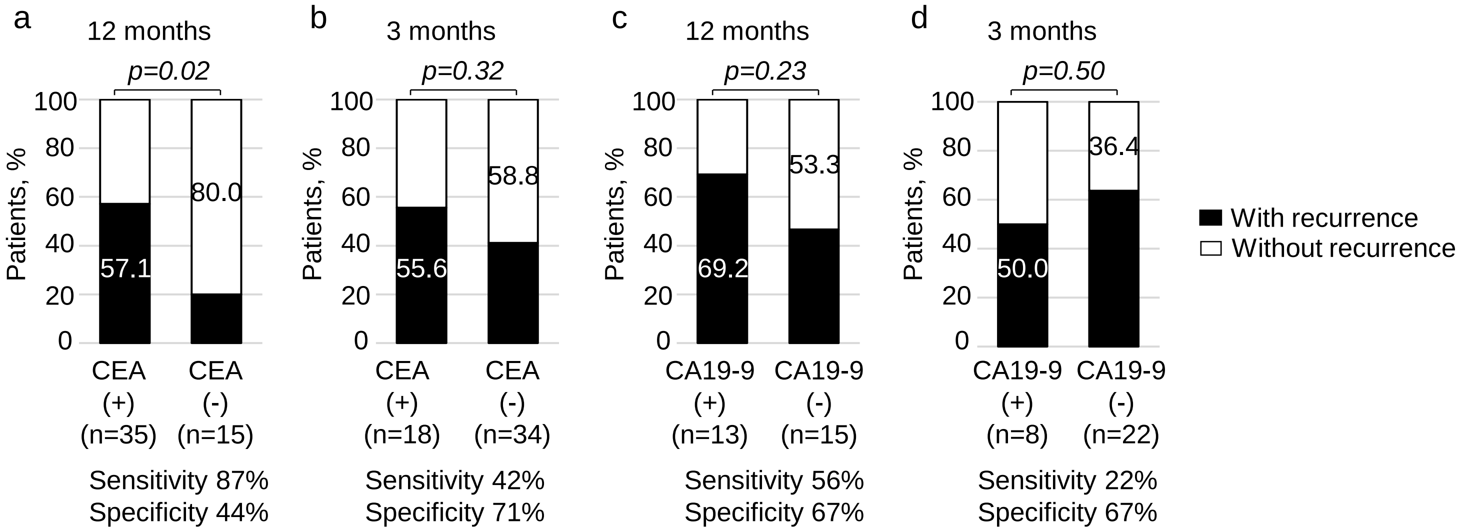

Percentages of patients with or without recurrence according to CEA status at 12 months (a) and 3 months (b), and CA19-9 status at 12 months (c) and 3 months (d) after surgery. *P* values were calculated using a two-sided chi-squared test comparing the distribution of factors between the two columns (positive vs negative) . The sensitivity and specificity for postoperative recurrence assessed by CEA or CA19-9 positivity are shown below the graph.

Supplementary Figure 5.

Recurrence rates based on the preoperative evaluation of serum NY-ESO-1 responses and adjuvant chemotherapy.

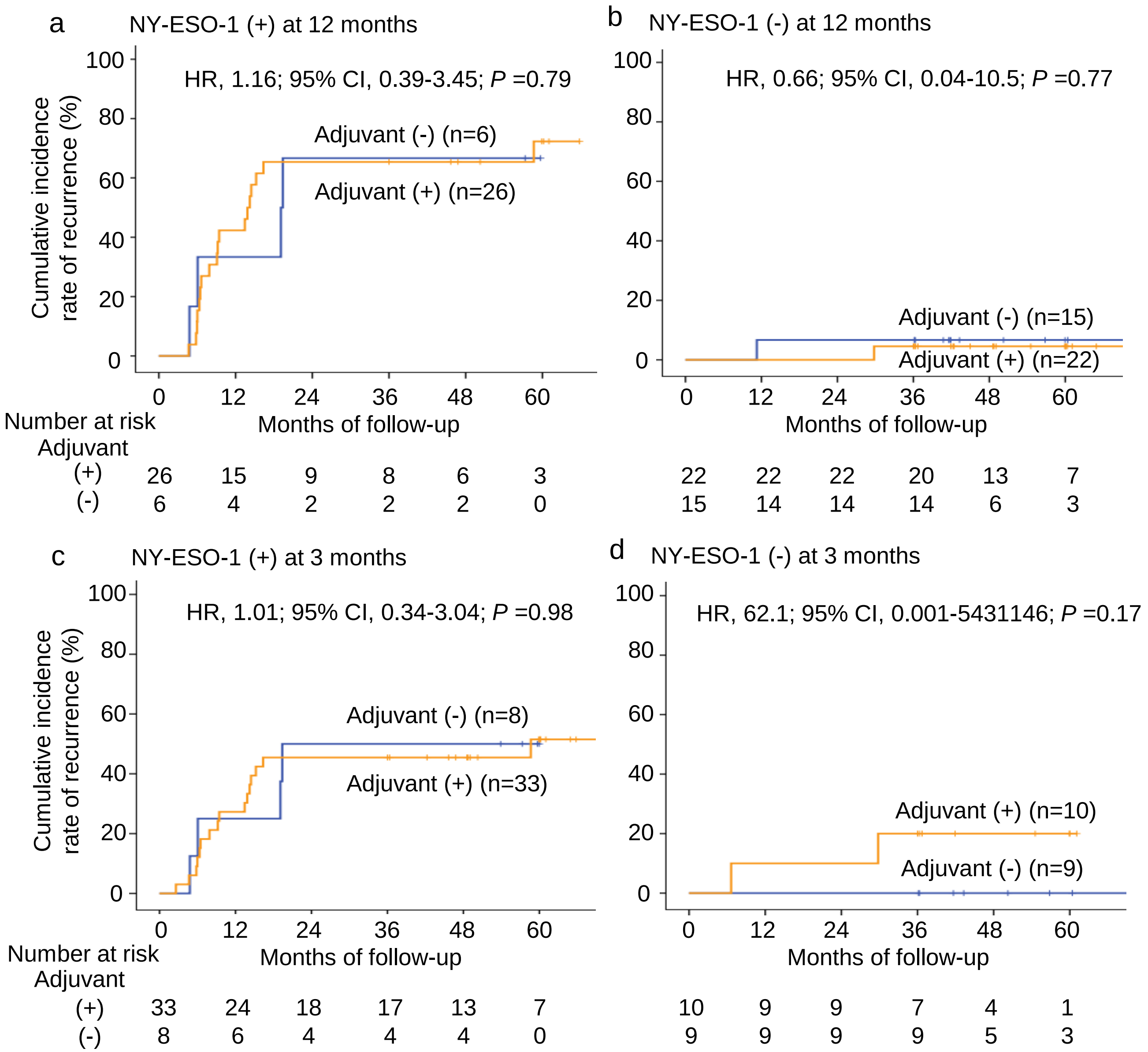

Kaplan-Meier analysis of cumulative recurrence rates was assessed by the administration of adjuvant chemotherapy according to the status of NY-ESO-1 antibody response at 12 months (a and b), and 3 months (c and d). HRs and 95% CIs were calculated using the Cox proportional hazard model. P values were calculated using the two-sided log-rank test.

Supplementary Figure 6.

Recurrence rates based on the preoperative evaluation of serum p53 responses and adjuvant chemotherapy.

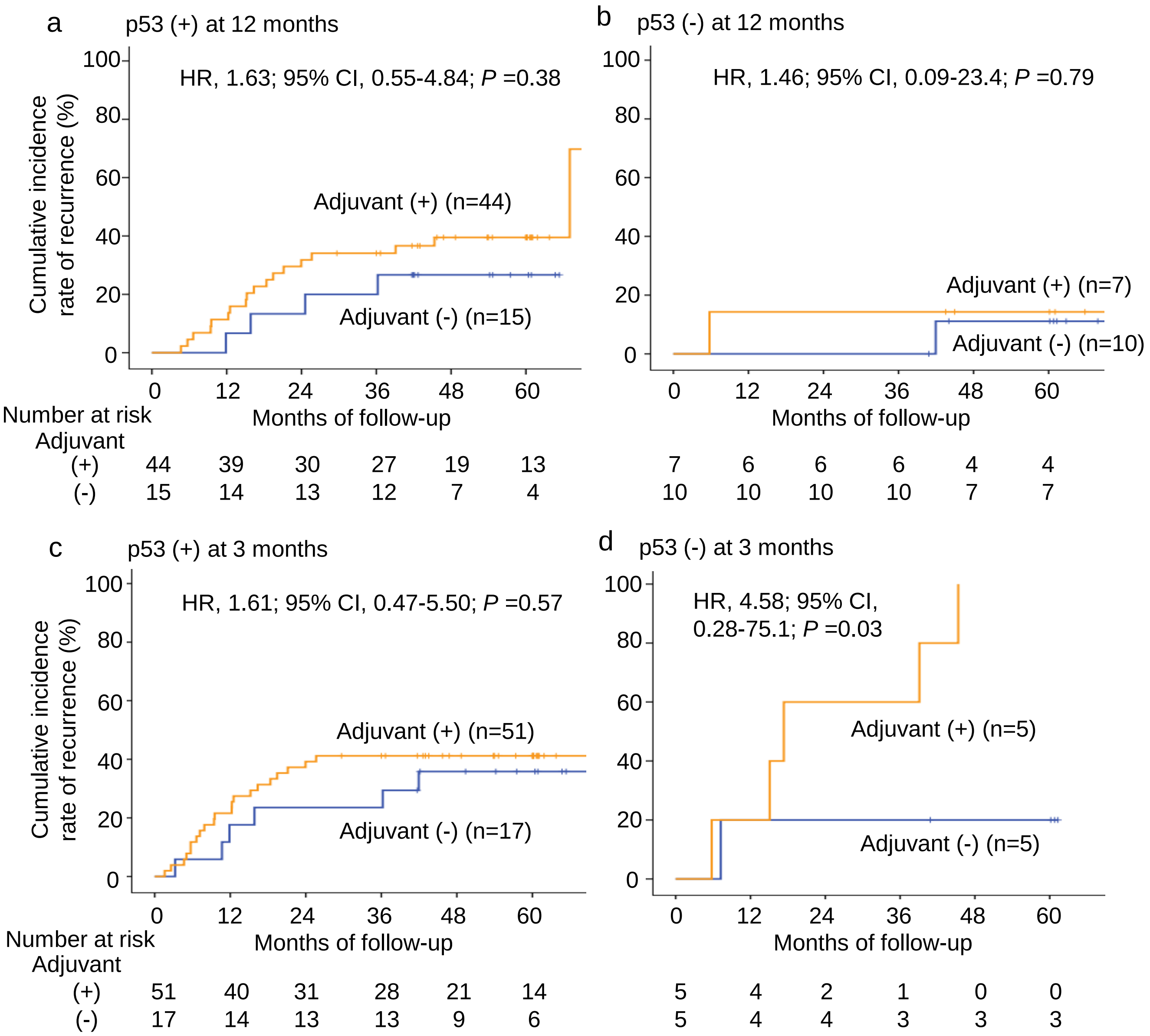

Kaplan-Meier analysis of cumulative recurrence rates was assessed by the administration of adjuvant chemotherapy according to the status of p53 antibody response at 12 months (a and b), and 3 months (c and d). HRs and 95% CIs were calculated using the Cox proportional hazard model. P values were calculated using the two-sided log-rank test.
